# Prediagnostic Blood Selenium Status and Mortality among Patients with Colorectal Cancer in Western European Populations

**DOI:** 10.3390/biomedicines9111521

**Published:** 2021-10-22

**Authors:** Jacqueline Roshelli Baker, Sushma Umesh, Mazda Jenab, Lutz Schomburg, Anne Tjønneland, Anja Olsen, Marie-Christine Boutron-Ruault, Joseph A. Rothwell, Gianluca Severi, Verena Katzke, Theron Johnson, Matthias B. Schulze, Giovanna Masala, Claudia Agnoli, Vittorio Simeon, Rosario Tumino, H. Bas Bueno-de-Mesquita, Inger Torhild Gram, Guri Skeie, Catalina Bonet, Miguel Rodriguez-Barranco, José María Houerta, Björn Gylling, Bethany Van Guelpen, Aurora Perez-Cornago, Elom Aglago, Heinz Freisling, Elisabete Weiderpass, Amanda J. Cross, Alicia K. Heath, David J. Hughes, Veronika Fedirko

**Affiliations:** 1 Department of Epidemiology, Rollins School of Public Health, Emory University, Atlanta, GA 30322, USA; jbaker21@alumni.emory.edu (J.R.B.); Sushma.umesh@gmail.com (S.U.); 2 International Agency for Research on Cancer, 69372 Lyon, France; jenabm@iarc.fr (M.J.); aglagoE@fellows.iarc.fr (E.A.); freislingh@iarc.fr (H.F.); Weiderpass@iarc.fr (E.W.); 3 Institut für Experimentelle Endokrinologie, Charité—Universitätsmedizin Berlin, CVK, Südring 10, 13353 Berlin, Germany; lutz.schomburg@charite.de; 4 Danish Cancer Society Research Center, Diet, Genes and Environment, Strandboulevarden 49, DK-2100 Copenhagen, Denmark; annet@cancer.dk (A.T.); anja@cancer.dk (A.O.); 5 CESP (UMR1018), Faculté de Médecine, Université Paris-Saclay, Inserm, Gustave Roussy, 94805 Villejuif, France; marie-christine.boutron@gustaveroussy.fr (M.-C.B.-R.); joseph.rothwell@gustaveroussy.fr (J.A.R.); gianluca.severi@inserm.fr (G.S.); 6 Department of Statistics, Computer Science and Applications (DISIA), University of Florence, 50123 Florence, Italy; 7 Division of Cancer Epidemiology, German Cancer Research Center (DKFZ), 69120 Heidelberg, Germany; v.katzke@dkfz-heidelberg.de (V.K.); t.johnson@dkfz.de (T.J.); 8 Department of Molecular Epidemiology, German Institute of Human Nutrition Potsdam-Rehbruecke, 14558 Nuthetal, Germany; mschulze@dife.de; 9 Institute of Nutrition Science, University of Potsdam, 14558 Nuthetal, Germany; 10 Cancer Risk Factors and Lifestyle Epidemiology Unit, Institute for Cancer Research, Prevention and Clinical Network-ISPRO, 50141 Florence, Italy; g.masala@ispro.toscana.it; 11 Epidemiology and Prevention Unit, Fondazione IRCCS Istituto Nazionale dei Tumori di Milano, 20133 Milan, Italy; claudia.agnoli@istitutotumori.mi.it; 12 Dipartimento di Salute Mentale e Fisica e Medicina Preventiva, Università degli Studi della Campania ‘Luigi Vanvitelli’, 80121 Naples, Italy; vittorio.simeon@unicampania.it; 13 Cancer Registry and Histopathology Department, Provincial Health Authority (ASP 7), 97100 Ragusa, Italy; rtuminomail@gmail.com; 14 Center for Nutrition and Health, National Institute for Public Health and the Environment, 3720 Bilthoven, The Netherlands; basbuenodemesquita@gmail.com; 15 Department of Community Medicine, The Arctic University of Norway, N-9037 Tromsø, Norway; inger.torhild.gram@uit.no (I.T.G.); guri.skeie@uit.no (G.S.); 16 Unitat de Nutrició i Càncer, 29010 Malaga, Spain; cbonet@iconcologia.net; 17 Escuela Andaluza de Salud Pública (EASP), Instituto de Investigación Biosanitaria ibs. Granada, 18014 Granada, Spain; miguel.rodriguez.barranco.easp@juntadeandalucia.es; 18 Centro de Investigación Biomédica en Red de Epidemiología y Salud Pública (CIBERESP), 28029 Madrid, Spain; jmhuerta.carm@gmail.com; 19 Department of Epidemiology, Murcia Regional Health Council, IMIB-Arrixaca, 30008 Murcia, Spain; 20 Department of Medical Biosciences, Umea University, 901 87 Umea, Sweden; bjorn.gylling@umu.se; 21 Department of Radiation Sciences, Umea University, 901 87 Umea, Sweden; bethany.vanguelpen@umu.se; 22 Cancer Epidemiology Unit, Nuffield Department of Population Health, University of Oxford, Oxford OX3 7LF, UK; aurora.perez-cornago@ndph.ox.ac.uk; 23 School of Public Health, Imperial College London, London SW7 2AZ, UK; amanda.cross@imperial.ac.uk (A.J.C.); a.heath@imperial.ac.uk (A.K.H.); 24 Cancer Biology and Therapeutics Group, School of Biomolecular and Biomedical Science, UCD Conway Institute, University College Dublin, D04 V1W8 Dublin, Ireland; 25 Department of Epidemiology, The University of Texas MD Anderson Cancer Center, Houston, TX 77030, USA

**Keywords:** selenium, selenoprotein P, colorectal cancer, survival, cohort

## Abstract

A higher selenium (Se) status has been shown to be associated with lower risk for colorectal cancer (CRC), but the importance of Se in survival after CRC diagnosis is not well studied. The associations of prediagnostic circulating Se status (as indicated by serum Se and selenoprotein P (SELENOP) measurements) with overall and CRC-specific mortality were estimated using multivariable Cox proportional hazards regression among 995 CRC cases (515 deaths, 396 from CRC) in the European Prospective Investigation into Cancer and Nutrition (EPIC) cohort. Se and SELENOP serum concentrations were measured on average 46 months before CRC diagnosis. Median follow-up time was 113 months. Participants with Se concentrations in the highest quintile (≥100 µg/L) had a multivariable-adjusted hazard ratio (HR) of 0.73 (95% CI: 0.52–1.02; P_trend_ = 0.06) for CRC-specific mortality and 0.77 (95% CI: 0.57–1.03; P_trend_ = 0.04) for overall mortality, compared with the lowest quintile (≤67.5 µg/L). Similarly, participants with SELENOP concentrations in the highest (≥5.07 mg/L) compared with the lowest quintile (≤3.53 mg/L) had HRs of 0.89 (95% CI: 0.64–1.24; P_trend_ = 0.39) for CRC-specific mortality and 0.83 (95% CI: 0.62–1.11; P_trend_ = 0.17) for overall mortality. Higher prediagnostic exposure to Se within an optimal concentration (100–150 µg/L) might be associated with improved survival among CRC patients, although our results were not statistically significant and additional studies are needed to confirm this potential association. Our findings may stimulate further research on selenium’s role in survival among CRC patients especially among those residing in geographic regions with suboptimal Se availability.

## 1. Introduction

Despite advances in prevention, screening, and treatment, colorectal cancer (CRC) remains the second most common cause of cancer death in Europe [1]. Currently, little is known about the effects of pre- and postdiagnostic dietary or lifestyle factors in CRC survival, with the only recognized prognostic factors of survival being tumor stage and grade. Several potentially modifiable factors related to diet and lifestyle have been suggested to be associated with survival among CRC patients [2,3]. However, research is limited especially on dietary micronutrients, including selenium.

Selenium (Se), an essential micronutrient, is a trace element that is involved in several major metabolic pathways and is thought to have anticarcinogenic properties [4]. The effects of Se are primarily mediated by selenoproteins, which have a variety of biological roles including modulation of redox homeostasis, antioxidant activity, thyroid metabolism, immune function, and inhibition of cell proliferation and angiogenesis [5,6,7]. Selenoprotein P (SELENOP) is a secreted glycoprotein that is predominantly produced by the liver and is considered the best biomarker of functional Se status [8]. SELENOP is a major transporter of hepatic Se in the blood, an indicator of longer-term Se intake [9], and may have local Se storage functions [10].

Observational and intervention studies suggest that blood Se status is associated with colorectal neoplasm risk, particularly in geographical regions with suboptimal Se availability due to low Se content in soils as in many European regions [11,12]. Furthermore, several -omics studies showed that Se status may influence the expression of genes and proteins implicated in antioxidant response, immune function, inflammatory pathways, cell growth and death, and cellular movement [13,14,15,16,17]. Additionally, emerging evidence from animal and cell culture studies supports the role of selenoproteins in improved CRC survival through their role in the regulation of programmed cell death and ability to inhibit angiogenesis [13,18]. These abilities have suggested the potential utility of Se compounds for cancer therapy [19,20]. However, several experimental studies showed that some selenoproteins (e.g., three important cellular redox-regulators: TXNRD1, SELENOF, and GPx2) may also promote malignant cell transformation and progression [5,21,22,23,24]. Hypoxic and oxidative stresses in proliferating tumors may decouple the normal hierarchy of selenoprotein expression [13,25].

The intake of Se and blood concentrations of Se and SELENOP vary significantly worldwide, with lower concentrations observed in the European population [26]. European and some Asian populations often exhibit a suboptimal blood Se status compared to North American populations where Se is more abundant in the soil and food system/diet [27]. The two large Se supplementation trials (the Selenium and Vitamin E Cancer Trial (SELECT) [28] and the Nutritional Prevention of Cancer (NPC) trial [29]) were conducted in the U.S., where 52% of the population take dietary supplements and have sufficient Se concentrations that could result in maximal selenoprotein activities or concentrations at baseline [30]. This, in part, could potentially explain why these trials demonstrated lower or no treatment efficacy of Se supplementation on primary outcomes of non-melanoma skin cancer incidence and prostate cancer risk [31]. Thus, it is important to understand the role of Se and its association with survival after cancer diagnosis in a population with relatively low exposure to Se.

Within this study, we investigated whether higher prediagnostic Se concentrations (within optimal range), as ascertained by circulating concentrations of Se and SELENOP, are associated with lower overall and CRC-specific mortality in patients diagnosed with CRC within the context of a large Western European prospective cohort study.

## 2. Materials and Methods

### 2.1. Study Population and Data Collection

The European Prospective Investigation into Cancer and Nutrition (EPIC) is a multicenter prospective cohort study designed to investigate the associations between diet, lifestyle, genetic and environmental factors, and various types of cancer. The rationale and methods of the EPIC design have been published previously [32,33]. Participating countries include France, Germany, Greece, Italy, The Netherlands, Spain, the UK, Sweden, Denmark, and Norway. Between 1992 and 1998, standardized dietary and lifestyle/personal history questionnaires, anthropometric data, and blood samples were collected from most participants at recruitment, before disease onset or diagnosis. Diet over the previous one year was measured at baseline by validated country-specific dietary questionnaires developed to ensure high compliance and better measures of local dietary habits. Serum samples were stored at the International Agency for Research on Cancer (IARC) at −196 °C in liquid nitrogen for all countries except Denmark (−150 °C, nitrogen vapor). Individuals who were eligible for the study were selected from the general population of a specific geographical area, town, or province. Exceptions included the French subcohort, which is based on members of the health insurance system or state-school employees; the Utrecht (The Netherlands) subcohort, which is based on women who underwent screening for breast cancer; and a portion of the Spanish and Italian subcohorts that included blood donors. The present analysis is based on participant data from all centers except for Norway (blood samples only recently collected; few CRCs diagnosed after blood donation), Sweden (no available serum samples), and Greece (excluded due to unforeseen data restriction issues). Written informed consent was provided by all study participants. Ethical approval for the EPIC study was obtained from the review boards of the IARC and local participating centers.

### 2.2. Cancer Incidence Follow-Up

Incident cancer cases were determined through record linkage with regional cancer registries (Denmark, Italy, The Netherlands, Spain, and the UK) or through a combination of methods including the use of health insurance records, contacts with cancer and pathology registries, and active follow-up through study subjects and their next-of-kin (France and Germany; complete up to June 2010).

### 2.3. Vital Status Follow-Up

Vital status follow-up was determined through record linkage with regional and/or national mortality registries (Denmark, Italy, The Netherlands, Spain, and the UK) or active follow-up (France and Germany). Censoring dates for complete follow-up varied amongst countries but were between December 2006 and October 2013 for France and Germany and between December 2006 and August 2013 for the remaining countries. Mortality was coded using the 10th Revision of the International Classification of Diseases, Injuries, and Causes of Death (ICD-10), and the outcome was assigned based on underlying cause of death. Twenty-four study participants had missing cause of death and were excluded only from the analysis of CRC-specific mortality. Exclusion of these 24 participants from the analysis of overall mortality did not change the results.

### 2.4. Case Ascertainment and Selection

Cancer data were coded using ICD-10 and the second revision of the International Classification of Disease for Oncology. CRC cases were selected from participants who developed colon (C18.0-C18.7), rectum (C19-C20), and overlapping or unspecified-origin colorectal tumors (C18.8-C18.9). CRC is defined as colon and rectal cancer cases. Of 1001 CRC cases with measurements of Se and SELENOP [12], 1 was excluded due to stage coded as in situ, and 5 cases were removed for having a follow-up time of 0, resulting in 995 CRC cases.

### 2.5. Selenium and Selenoprotein P Measurements

Information regarding prior collection and measurement of serum Se and SELENOP as part of a previously conducted nested case–control study on CRC has been published [12]. Briefly, total Se concentrations were measured from 20 µL samples of blood serum using X-ray fluorescence spectrometer (Picofox^TM^ S2, Bruker Nano GmbH, Berlin, Germany). SELENOP concentrations were measured from 20 µL blood serum samples and quantified by conducting an immunoluminometric sandwich assay (Selenotest^TM^, ICI GmbH, Berlin, Germany). All sample measurements were done in duplicate, and mean concentration values were used in the analysis. Se measurements were controlled with a commercial standard serum (Seronorm, Billingstad, Norway) and an atomic absorption standard (1000 mg/mL, Sigma, Taufkirchen, Germany). The coefficients of variation (CVs) were 7.3% and 7.2% for SELENOP controls of 1.5 and 8.6 mg/L, respectively.

### 2.6. Covariates

The following a-priori-identified covariates were collected at baseline and assessed as potential confounders: age at diagnosis, sex, tumor stage, grade of tumor differentiation (well, moderately, poorly, unknown), location of primary tumor (colon or rectum), smoking status (never smoker, former smoker, current smoker, unknown), body mass index (BMI, kg/m^2^), physical activity (combined recreational and household activity as measured by the Cambridge index; expressed as sex-specific categories of metabolic equivalents), year of diagnosis, several dietary components (intakes of red and processed meats, alcohol, fish and shellfish, nuts and seeds, fruits and vegetables, and total energy), Healthy Lifestyle Index score [34], Mediterranean diet score [35], and baseline self-reported diabetes status. Healthy Lifestyle Index score and Mediterranean diet score were adjusted for in separate models. These variables were chosen based on previous published evidence showing their associations with CRC incidence or survival and/or blood Se concentrations. Information regarding categorization and harmonization of tumor stage data has been previously published [36]. Confounding assessment was conducted by evaluating whether there was a >10% change in hazard ratios (HRs) after including the variable in the model. Age at diagnosis, sex, stage, location of primary tumor, smoking status, BMI (kg/m^2^), year of diagnosis, and baseline diabetes status were included in the final analysis.

### 2.7. Statistical Analyses

Death from CRC was the primary endpoint, and death from any cause was used as a secondary endpoint. Age at first tumor diagnosis and age at death or censorship were used as the two time interval points for patient follow-up time. Participants were counted as censored if they immigrated or were lost to follow-up. Separate categories were created for categorical variables with missing values. Adjusted cumulative incidence curves were used to assess the association of Se and SELENOP concentrations on CRC-specific mortality accounting for competing risks (deaths from other causes) [37]. To evaluate the association between Se and SELENOP concentrations and CRC-death and overall mortality, Cox proportional hazards models stratified by country and adjusted for sex, stage of tumor, BMI, smoking status, location of tumor, year of diagnosis, age of diagnosis, and baseline diabetes were used to calculate hazard ratios (HRs) and 95% confidence intervals (CIs). The proportional hazards assumption was graphically assessed by estimating “log–log” survival curves and checked for parallelism. In addition, the proportional hazards assumption was verified using goodness of fit test methods. Correlations between Schoenfeld residuals and time-dependent variables in the Cox model were evaluated to test for any violations of the proportional hazard assumptions. The exposure was examined as follows: Se and SELENOP quintiles, per 21.99 µg/L (one standard deviation) increase of Se and per 0.96 mg/L (one standard deviation) increase of SELENOP. We also considered sex-specific quintiles but do not show them here, as the results were very similar to those reported. *p*-value for trend was calculated with the median value of each Se and SELENOP quintile included as a continuous variable in the corresponding models.

We explored whether the association between Se or SELENOP and risk for CRC-specific and overall mortality is non-linear using non-parametric restricted cubic splines [38,39] fitted to a Cox proportional hazards model using the SAS macro “lgtphcurv9” [40]. Tests for non-linearity used the likelihood ratio test, comparing the model with only the linear term to the model with the linear and cubic spline terms [40]. *p*-values of non-linearity tests from these models were consistent with a linear response (Supplementary Figures S1–S4).

The effect of missing tumor stage information on effect estimates was assessed using several approaches. The first approach reclassified missing tumor stage values into a separate missing category and adjusted for the stage variable in the final model (included in the primary analysis). Second, a sensitivity analysis was conducted by excluding participants with missing stage information and subsequently by assessing how the results were affected by the missing stage information. Finally, an imputation of missing tumor stage values was conducted using the SAS PROC MI procedure [41]. The multiple imputation method was based on available data for the other covariates in the model and assumed that the stage data were missing at random.

Subgroup analyses by categories of potentially biologically relevant effect modifiers (length of follow-up, sex, age at diagnosis, tumor site, tumor grade, tumor stage, year of diagnosis, smoking status, and BMI) were conducted. We also stratified by geographic region categorized as Northern (Denmark), Central (UK, The Netherlands, Germany, North of France), and Southern (South of France, Italy, Spain). Stratified multivariable-adjusted HRs and 95% CIs were reported per one SD increase in Se or SELENOP. A cross-product of Se or SELENOP as a continuous variable and the covariate of interest as a continuous or categorical variable was included in the model to test for multiplicative statistical interaction; and the likelihood ratios based on the models with and without the interaction terms were used to test for statistical significance.

All statistical tests were conducted using SAS version 9.2 (SAS Institute), and *p*-values of <0.05 were considered statistically significant.

## 3. Results

### 3.1. Characteristics of Study Participants

The distribution of selected baseline characteristics of CRC cases according to quintiles of serum Se are shown in Table 1 (similar distributions were observed according to quintiles of SELENOP and, thus, were not shown). Among 995 eligible CRC cases, there were 515 deaths (including deaths from CRC = 396, other malignant neoplasms = 43, cardiovascular disorders = 23, several other causes with low frequency in each category = 29, and missing cause of death = 24). Two participants were excluded from Se analysis (N = 993) and five participants were excluded from SELENOP analysis (N = 990) due to missing values. Median follow-up time was 113 months (standard deviation, SD = 70, 25th percentile = 22, 75th percentile = 159), and Se and SELENOP were measured on average 46 months (SD = 29, 25th percentile = 25, 75th percentile = 65) before CRC diagnosis. The Se concentration range was 29 to 142 µg/L, below concentrations associated with acute toxicity (>400 μg/L).

### 3.2. Selenium and Mortality among CRC Patients

The results of age, sex, stage-adjusted, and multivariable-adjusted Cox proportional hazard models for the association of Se with CRC-specific and overall mortality are shown in Table 2. Higher concentrations of Se were associated with lower CRC-specific mortality and overall mortality, although these observations were not statistically significant. For CRC-specific mortality, the multivariable adjusted HR for the fifth quintile versus the first quintile of Se concentration was 0.73 (95% CI: 0.52–1.02, *P*_trend_ = 0.06). The HR per one SD increase in Se concentration was 0.90 (95% CI: 0.81–1.00). There also was a suggestive inverse association between Se concentrations and overall mortality, where the HR for the fifth quintile versus the first quintile was 0.77 (95% CI: 0.57–1.03, *P*_trend_ = 0.04). The HR per one SD increase in Se concentration was 0.91 (95% CI: 0.83–1.00). In a sensitivity analysis, restricting to cases with complete stage data resulted in HRs of 0.92 (95% CI: 0.82–1.03) and 0.91 (95% CI: 0.82–1.00) for CRC-specific and overall mortality, respectively (Table 3). Similar results were obtained with imputed CRC stage data analyses for CRC-specific (HR = 0.91, 95% CI: 0.82–1.02) and overall mortality (HR = 0.92, 95% CI: 0.84–1.01; Table 3) and accounting for competing risks of death for CRC-specific mortality (Supplementary Figure S5).

### 3.3. Selenoprotein P and Mortality among CRC Patients

The results of age, sex, stage-adjusted, and multivariable-adjusted Cox proportional hazard models for the associations of SELENOP and CRC-specific and overall mortality are shown in Table 2. SELENOP concentration was inversely but not statistically significantly associated with CRC-specific mortality: HR for the fifth quintile versus the first quintile was 0.89 (95% CI: 0.64–1.24, *P*_trend_ = 0.39). Higher concentrations of SELENOP were also not associated with a statistically significant reduction in overall mortality: HR for the fifth quintile versus the first quintile was 0.83 (95% CI: 0.62–1.11, *P*_trend_ = 0.17). In a sensitivity analysis, restricting to cases with complete stage data resulted in HRs of 0.95 (95% CI: 0.86–1.06) and 0.94 (95% CI: 0.84–1.04) for CRC-specific and overall mortality, respectively (Table 3). Similar results were obtained with imputed CRC stage data analyses for CRC-specific (HR = 0.97, 95% CI: 0.87–1.08) and overall mortality (HR = 0.94, 95% CI: 0.86–1.04; Table 3) and accounting for competing risks of death for CRC-specific mortality (Supplementary Figure S6).

### 3.4. Stratified Analyses

There were no substantial differences in associations between Se or SELENOP and CRC-specific or overall mortality across select subcategories of potential a-priori-defined biologically plausible effect modifiers (Table 3). However, regional groupings showed stronger significant protective associations between serum Se and overall and CRC-specific mortality in the Northern European region compared to the Central and Southern European regions (*P*_interaction_ = 0.03 and *P*_interaction_ = 0.01, respectively). There was a suggestion of a stronger protective association between serum Se concentrations and overall and CRC-specific mortality among CRC cases with advanced disease (stage III–IV; *P*_interaction_ = 0.11 and *P*_interaction_ = 0.10, respectively). Furthermore, there was a suggestion of a stronger protective association between serum Se concentrations and overall and CRC-specific mortality among CRC cases diagnosed with CRC within five years of blood collection (*P*_interaction_ = 0.01 and *P*_interaction_ = 0.03, respectively).

## 4. Discussion

This study is the first prospective analysis of the association of prediagnostic serum Se status biomarkers with mortality among CRC patients. The results of this study suggest that higher prediagnostic total serum Se and SELENOP concentrations may be associated with lower mortality among patients with CRC in Western Europe; however, the effect estimates were statistically non-significant. This indicates that prediagnostic Se status might be a potential factor affecting survival in CRC patients, particularly from a population with low Se status, such as in Europe [31], but larger studies including those in other settings are needed to confirm these results.

Strong basic science experimental evidence and data from epidemiologic studies of Se status and CRC risk support a possible association between the micronutrient and survival after CRC diagnosis [5]. However, there were no previous observational studies investigating this association. A recent Cochrane systematic review [42] reported that Se supplementation did not reduce overall cancer incidence (relative risk [RR] = 0.99, 95% CI: 0.86–1.14) or mortality (RR = 0.81, 95% CI: 0.49–1.32) among individuals included in five randomized controlled trials (RCTs). However, compared with the lowest category, the highest category of Se exposure, as measured by Se blood concentrations or dietary intake, was associated with lower cancer incidence (summary odds ratio [OR] = 0.72, 95% CI: 0.55–0.93) and lower cancer mortality; OR = 0.76, 95% CI: 0.59–0.97) in 14 observational studies [42]. The discrepancy between the findings of the RCTs and the observational studies could be due to differences in study populations with regard to their baseline Se status, type of study population, and the chemical form and/or source of Se (high-dose supplementation [43] versus dietary intake). Furthermore, the review did not focus on cancer survivors.

Interestingly, findings from observational studies on several other tumor types support an association of higher Se intake/status with better survival outcomes. A study of 3146 women diagnosed with invasive breast cancer in the Swedish Mammography Cohort reported an inverse association between dietary Se intake and breast cancer mortality (Q_4_ vs. Q_1_: HR = 0.64, 95% CI: 0.48–0.84) [44]. Another study showed that low Se blood concentrations were associated with higher risk of death in 546 women with breast cancer (<64.4 vs. >81 μg/L: HR = 2.49, 95% CI: 1.53–4.04) [45]. A small study of 41 renal cancer patients in Germany suggested that lower SELENOP serum concentrations may be associated with poor survival [46]. Another study among 302 lung cancer patients in Poland also found that low serum Se concentrations were associated with a higher risk of death, particularly among patients with stage I disease (<57.9 vs. >69.3 μg/L: HR = 2.73, 95% CI: 1.21–6.11) [47]. Similarly, low serum Se concentrations were associated with worse outcomes among 296 laryngeal cancer patients in a prospective study in Poland (<50 vs. >67 μg/L: HR = 3.07, 95% CI: 1.59–5.94) [48]. In the above studies [45,46,47,48], Se status was assessed at the time of diagnosis, whereas no previous studies considered prediagnostic exposure to Se, which may contribute to lower tumor aggressiveness and its metastatic potential. Our results for CRC are in line with these previously published reports for other cancers [45,46,47,48] and suggest a potential inverse association, with high Se status before CRC diagnosis associated with improved survival in CRC patients from Western European populations with relatively low exposure to Se. Our results also suggested that this association might be stronger in the Northern European region compared to the Central and Southern regions. Although only one country—Denmark—was included in the Northern region, limiting the interpretability of this finding, it was characterized by statistically significantly higher Se concentrations (age- and sex-adjusted mean = 90.6, 95% CI: 88.3–93.0 µg/L) compared to the Central (age- and sex-adjusted mean = 79.5, 95% CI: 77.4–81.5 µg/L) and Southern (age- and sex-adjusted mean = 84.8, 95% CI: 81.9–87.6 µg/L) regions (all *p*-values <0.002).

Selenoproteins have been implicated in the regulation of multiple cell signaling pathways, many of which have been linked to colorectal neoplasm development and progression [13]. Several selenoproteins expressed in colorectal tissue have well-established functions in redox control and response to oxidative stress and inflammation, which are hallmark processes in colorectal carcinogenesis [13]. Selenoproteins may also prevent cancer progression due to their role in the regulation of programmed cell death and the cell cycle, and inhibition of cellular proliferation [13,18,44]. Furthermore, Se supplementation was shown to inhibit microvascular development [49], and affect the vascular endothelial growth factor (VEGF), and thereby inhibit angiogenesis [50]. Se can also downregulate the expression of several genes including those involved in osteopontin and collagen metabolism. This downregulation may have antimetastatic effects [51,52]. Se has also been reported to suppress glutaminolysis, a biochemical reaction responsible for energy production in tumor cells [53]. It has been proposed that altered selenoprotein expression in the colorectal tract due to limited Se supply could increase cancer risk by weakening the gut epithelial cell response to harmful oxidative and inflammatory challenges [54]. However, several studies also indicated that some selenoproteins, namely important cellular redox regulators TXNRD1, SELENOF, and GPx2, may both prevent and promote cancer [5,55]. Since these oxidoreductase functions are needed by both normal and cancer cells, they could also result in anti- and pro-tumorigenic effects at a tissue-specific cellular level and be dependent on the tumor stage/grade and Se availability [5,25,54]. Hypoxic and oxidative stresses in proliferating tumors may modify selenoprotein expression during carcinogenesis [13,25].

Strengths of this study include the large prospective design and the measurement of both serum Se and SELENOP concentrations, the latter likely being the most informative parameter of Se status. In addition, the European population has shown a range of Se status concentrations [12] from suboptimal (majority of the subjects) to replete, making this population a suitable population for this analysis. Specifically, 96% of the EPIC subjects had serum Se concentrations below 124 µg/L, and, therefore, they can be deemed to have a suboptimal Se intake, as Se intake ideally should provide a circulating Se concentration of ≥124 ug/L for maximal expression of the SELENOP and glutathione peroxidase 3 (GPx3) selenoproteins, as a measure of Se sufficiency [11]. Selenoprotein expression is affected by Se availability and other factors such as genetics, sex, health status, and disease-related conditions [56,57,58,59]. Furthermore, we were able to control for multiple potential confounders and accounted for missing information on CRC stage through various techniques including sensitivity analyses and imputation techniques.

However, there were several limitations in this study. First, we did not have information on CRC treatment received by the participants, which may influence CRC outcome. To address this issue, we conducted our analyses by stratifying on the basis of the country of CRC diagnosis, while adjusting for year of diagnosis and tumor stage as a proxy for treatment. Second, we were only able to measure Se concentrations once. Prior research, however, supports a strong correlation between blood Se concentrations and long-term Se intake [60]. As with other observational studies, there is the possibility for residual confounding despite controlling for relevant covariates. However, in our multivariable models, many potentially important confounding variables for CRC survival were considered. It is also important to note that cancer survivors are very likely to make lifestyle changes including initiation of vitamin and mineral supplement use after cancer diagnosis. Therefore, the prediagnostic measurements are more reflective of the environment in which tumors develop, and it might be possible that cancer in Se-deficient individuals is more aggressive/lethal [61]. In our data, there was a suggestion of a stronger protective association between Se concentrations and overall and CRC-specific mortality among CRC cases with advanced disease (stage III/IV), supporting the possible influence of Se on tumor molecular phenotype at later stages. It is also possible that higher Se/SELENOP concentrations are acting as a proxy for a healthy lifestyle (e.g., healthier diet), which may independently influence CRC survival. However, our results were adjusted for prediagnostic BMI, and adjustments for dietary intakes did not change the study results. In the stratified analyses, the inverse associations of Se and SELENOP with mortality were limited to CRC cases diagnosed within five years of blood collection. This could indicate that either the tumor influences Se concentrations or that Se-deficient individuals have more aggressive tumors or a poorer overall health. Finally, due to geographical differences in Se soil content and hence in the food system, results from this type of study may be difficult to generalize to a population with sufficient Se concentrations. However, it allowed us to have a wide range of Se concentrations in our study.

## 5. Conclusions

In summary, the findings from this study suggest a statistically non-significant inverse association between prediagnostic Se status and overall and CRC-specific mortality among CRC patients in a population that largely has suboptimal Se status. Further research is necessary to replicate these findings in different populations and to understand the mechanisms of action of Se metabolism in relation to tumor development and progression.

## Figures and Tables

**Table 1:** Selected baseline characteristics of CRC cases according to quintile of prediagnostic serum Se in the EPIC study.

		Selenium, µg/L				
	Characteristic ^a^	Quintile 1	Quintile 2	Quintile 3	Quintile 4	Quintile 5
		≤67.5	67.6–77.4	77.5–88.0	88.1–99.9	≥100.0
		(N = 197)	(N = 201)	(N = 198)	(N = 199)	(N = 198)
Selenium (µg/L), mean (SD)		56.7 (9.0)	72.6 (2.9)	82.6 (2.8)	94.2 (3.4)	115.6 (18.4)
Selenoprotein P (mg/L), mean (SD)		3.5 (0.8)	4.1 (0.8)	4.3 (0.7)	4.6 (0.7)	5.1 (0.9)
Age at diagnosis (years), mean (SD)		62.6 (7.8)	61.7 (7.1)	62.5 (7.1)	62.0 (7.6)	62.7 (7.0)
BMI (kg/m^2^), mean (SD)		26.4 (4.4)	26.5 (4.3)	26.8 (4.4)	26.8 (4.6)	26.9 (4.2)
Women, N (%)		117 (59.4)	114 (56.7)	100 (50.5)	94 (47.2)	91 (46.0)
Location of primary tumor, N (%)						
	Colon	139 (70.6)	134 (66.7)	123 (62.1)	103 (51.8)	127 (64.1)
	Rectum	58 (29.4)	67 (33.3)	75 (37.9)	96 (48.2)	71 (35.9)
Stage of tumor, N (%)						
	I	28 (14.2)	53 (26.4)	38 (19.2)	39 (19.6)	35 (17.7)
	II	52 (26.4)	37 (18.4)	43 (21.7)	45 (22.6)	43 (21.7)
	III	62 (31.5)	52 (25.9)	67 (33.8)	63 (31.7)	77 (38.9)
	IV	24 (12.2)	31 (15.4)	24 (12.1)	25 (12.6)	19 (9.6)
Tumor grade, N (%)						
	Well differentiated	11 (5.6)	8 (4.0)	15 (7.6)	9 (4.5)	7 (3.5)
	Moderately differentiated	69 (35.0)	73 (36.3)	57 (28.8)	51 (25.6)	34 (17.2)
	Poorly differentiated	18 (9.1)	12 (6.0)	13 (6.6)	17 (8.5)	10 (5.1)
	Unknown	99 (50.3)	108 (53.7)	113 (57.1)	122 (61.3)	147 (74.3)
Smoking status, N (%)						
	Never smoker	80 (40.6)	83 (41.3)	79 (39.9)	80 (40.2)	72 (36.4)
	Former smoker	60 (30.5)	56 (27.9)	69 (34.9)	66 (33.2)	72 (36.4)
	Current smoker	56 (28.4)	60 (29.9)	49 (24.8)	52 (26.1)	54 (27.3)
Physical activity, N (%)						
	Inactive	27 (13.7)	35 (17.4)	34 (17.2)	29 (14.6)	34 (17.2)
	Moderately inactive	59 (30.0)	52 (25.9)	58 (29.3)	61 (30.7)	64 (32.3)
	Moderately active	92 (46.7)	87 (43.3)	84 (42.4)	89 (44.7)	83 (41.9)
	Active	17 (8.6)	24 (11.9)	22 (11.1)	20 (10.1)	17 (8.6)
Self-reported diabetes, N (%)						
	No	148 (75.1)	156 (77.6)	167 (84.3)	151 (75.9)	146 (73.7)
	Yes	13 (6.6)	6 (3.0)	6 (3.0)	9 (4.5)	15 (7.6)
Alcohol (grams/day), mean (SD)		17.2 (24.5)	15.8 (19.7)	20.3 (23.3)	17.9 (21.1)	19.5 (21.9)
Overall mortality, N (%)		103 (52.3)	104 (51.7)	104 (52.5)	102 (51.3)	101 (51.0)
CRC-specific mortality, N (%)		83 (42.1)	82 (40.8)	79 (39.9)	76 (38.2)	75 (37.9)

^a^ Unknown/missing values of categorical variables were classified as a separate category: smoking status (N = 5), diabetes (N = 176), physical activity (N = 5), stage of tumor (N = 136). Percentages may not add up to 100% in each category since unknown values were not excluded from the frequency calculations.

**Table 2:** HRs and 95% CIs for overall and CRC-specific mortality according to quintiles of prediagnostic serum Se and SELENOP among CRC cases in the EPIC study.

Selenium				Selenoprotein P		
	Deaths/Total	µg/L	HR (95% CI)	Deaths/Total	mg/L	HR (95% CI)
**Overall mortality**						
Age-, Sex-, Stage-adjusted ^a^						
Quintile 1	103/197	≤67.5	1.00 (ref)	109/197	≤3.53	1.00 (ref)
Quintile 2	104/201	67.6–77.4	1.07 (0.80–1.42)	104/199	3.54–4.06	0.96 (0.72–1.28)
Quintile 3	104/198	77.5–88.0	0.91 (0.68–1.22)	95/199	4.07–4.50	0.77 (0.57–1.02)
Quintile 4	102/199	88.1–99.9	0.91 (0.68–1.22)	91/197	4.51–5.06	0.75 (0.56–1.00)
Quintile 5	101/198	≥100.0	0.76 (0.56–1.02)	114/198	≥5.07	0.87 (0.65–1.15)
*P*_trend_ ^b^			0.04			0.27
per one SD ^c^	514/993		0.91 (0.83–1.00)	513/990		0.95 (0.86–1.04)
Multivariable-adjusted ^d^						
Quintile 1	103/197	≤67.5	1.00 (ref)	109/197	≤3.53	1.00 (ref)
Quintile 2	104/201	67.6–77.4	1.05 (0.79–1.41)	104/199	3.54–4.06	1.00 (0.75–1.33)
Quintile 3	104/198	77.5–88.0	0.95 (0.71–1.27)	95/199	4.07–4.50	0.80 (0.59–1.07)
Quintile 4	102/199	88.1–99.9	0.93 (0.69–1.25)	91/197	4.51–5.06	0.74 (0.55–1.00)
Quintile 5	101/198	≥100.0	0.77 (0.57–1.03)	114/198	≥5.07	0.83 (0.62–1.11)
*P*_trend_ ^b^			0.04			0.17
per one SD ^c^	514/993		0.91 (0.83–1.00)	513/990		0.94 (0.85–1.03)
**CRC-specific mortality ^e^**						
Age-, Sex-, Stage-adjusted ^a^						
Quintile 1	83/192	≤67.5	1.00 (ref)	87/193	≤3.53	1.00 (ref)
Quintile 2	82/198	67.6–77.4	1.06 (0.76–1.47)	78/191	3.54–4.06	0.99 (0.71–1.37)
Quintile 3	79/192	77.5–88.0	0.93 (0.67–1.30)	70/194	4.07–4.50	0.73 (0.52–1.03)
Quintile 4	76/194	88.1–99.9	0.88 (0.63–1.23)	69/196	4.51–5.06	0.70 (0.50–0.99)
Quintile 5	75/193	≥100.0	0.73 (0.52–1.03)	91/192	≥5.07	0.92 (0.66–1.27)
*P*_trend_ ^b^			0.07			0.48
per one SD ^c^	395/969		0.91 (0.81–1.01)	395/966		0.96 (0.86–1.07)
Multivariable-adjusted ^d^						
Quintile 1	83/192	≤67.5	1.00 (ref)	87/193	≤3.53	1.00 (ref)
Quintile 2	82/198	67.6–77.4	1.05 (0.76–1.46)	78/191	3.54–4.06	1.04 (0.74–1.44)
Quintile 3	79/192	77.5–88.0	0.95 (0.68–1.32)	70/194	4.07–4.50	0.76 (0.54–1.07)
Quintile 4	76/194	88.1–99.9	0.88 (0.63–1.24)	69/196	4.51–5.06	0.71 (0.50–1.00)
Quintile 5	75/193	≥100.0	0.73 (0.52–1.02)	91/192	≥5.07	0.89 (0.64–1.24)
*P*_trend_ ^b^			0.06			0.39
per one SD ^c^	395/969		0.90 (0.81–1.00)	395/966		0.95 (0.86–1.06)

^a^ Adjusted for age, sex, and stage; stratified by country. ^b^ *P*_trend_ was calculated using the median value of each Se or SELENOP quintile included as a continuous variable, adjusted for variables in the corresponding models. ^c^ One SD = 21.99 µg/L Se; one SD = 0.96 mg/L of SELENOP. ^d^ Adjusted for age, sex, stage, smoking status, BMI, site of primary tumor, year of diagnosis, and baseline diabetes; stratified by country. ^e^ Excluded 24 cases with missing cause of death.

**Table 3:** Multivariable-adjusted HRs and 95% CIs for an increment of one SD of Se or SELENOP for overall and CRC-specific mortality in sensitivity analyses and across strata of potential effect modifiers among CRC cases in the EPIC study.

			Overall Mortality		CRC-Specific Mortality ^a^		
Risk Factor		Deaths/Total	HR (95% CI) ^b^	*P*_trend_ ^d^ _or interaction_ ^c^	Deaths/Total	HR (95% CI) ^b^	*P*_trend_ ^d^ _or interaction_ ^c^
**Selenium**							
All participants		514/993	0.91 (0.83–1.00)	0.04 ^d^	395/969	0.90 (0.81–1.00)	0.06 ^d^
Sensitivity analyses							
Complete CRC stage data ^e^		450/857	0.91 (0.82–1.00)	0.05 ^d^	355/839	0.92 (0.82–1.03)	0.14 ^d^
Imputed CRC stage data		514/993	0.92 (0.84–1.01)	0.07 ^d^	395/969	0.91 (0.82–1.02)	0.09 ^d^
Follow-up (years)							
	≥2	257/732	0.94 (0.83–1.07)	0.37 ^d^	165/711	0.94 (0.80–1.10)	0.42 ^d^
	≥4	158/631	1.02(0.87–1.19)	0.83 ^d^	77/611	1.03 (0.84–1.28)	0.76 ^d^
Time between blood collection and diagnosis (years)							
	<2.6	176/330	0.93 (0.78–1.12)	0.03	138/325	0.89 (0.73–1.10)	0.01
	[2.6–4.8)	175/332	0.86 (0.73–1.01)		133/321	0.84 (0.70–1.02)	
	≥4.8	163/331	1.00 (0.83–1.21)		124/323	1.00 (0.80–1.25)	
Stratified Analyses							
Sex							
	Women	252/516	0.92 (0.80–1.06)	0.52	202/505	0.93 (0.79–1.08)	0.48
	Men	262/477	0.87 (0.77–1.00)		193/464	0.86 (0.73–1.01)	
Age at diagnosis (years)							
	<62.4	215/497	0.94 (0.80–1.11)	0.51	184/493	0.95 (0.80–1.13)	0.38
	≥62.4	299/496	0.89 (0.79–1.00)		211/476	0.86 (0.75–0.99)	
Cancer site							
	Colon	333/626	0.89(0.80–1.00)	0.56	255/611	0.89 (0.78–1.01)	0.87
	Rectum	181/367	0.92 (0.77–1.09)		140/358	0.90 (0.74–1.09)	
Stage ^e^							
	I–II	128/413	1.03 (0.84–1.25)	0.11	79/399	1.08 (0.85–1.37)	0.10
	III–IV	322/444	0.90 (0.80–1.01)		276/440	0.89 (0.78–1.01)	
Region ^f^							
	Northern	194/330	0.79 (0.68–0.91)	0.03	158/325	0.75 (0.64–0.89)	0.01
	Central	227/436	0.97 (0.83–1.13)		163/418	0.94 (0.79–1.13)	
	Southern	93/227	1.03 (0.81–1.30)		74/226	1.21 (0.92–1.58)	
**Selenoprotein P**							
All participants		513/990	0.94 (0.85, 1.03)	0.17 ^d^	395/966	0.95 (0.86–1.06)	0.39 ^d^
Sensitivity analyses							
Complete CRC stage data ^e^		450/856	0.94 (0.84, 1.04)	0.22 ^d^	355/838	0.95 (0.86–1.06)	0.39 ^d^
Imputed CRC stage data		513/990	0.94 (0.86, 1.04)	0.24 ^d^	395/966	0.97 (0.87–1.08)	0.58 ^d^
Follow-up (years)							
	≥2	257/730	0.93 (0.82, 1.07)	0.31 ^d^	165/709	0.97 (0.82–1.15)	0.74 ^d^
	≥4	158/629	0.99 (0.83, 1.17)	0.88 ^d^	77/609	1.10 (0.86–1.40)	0.45 ^d^
Time between blood collection and diagnosis (years)							
	<2.6	176/329	0.88 (0.74–1.05)	0.15	138/324	0.88 (0.72–1.07)	0.45
	[2.6–4.8)	175/331	0.87(0.74–1.04)		133/320	0.90 (0.74–1.10)	
	≥4.8	162/330	1.06(0.88–1.28)		124/322	1.16 (0.93–1.44)	
Stratified Analyses							
Sex							
	Women	252/514	0.91(0.79–1.06)	0.74	202/503	0.97 (0.82–1.15)	0.76
	Men	261/476	0.92(0.81–1.05)		193/463	0.92 (0.79–1.08)	
Age at diagnosis (years)							
	<62.4	216/497	0.91(0.78–1.07)	0.66	185/493	0.91 (0.76–1.08)	0.83
	≥62.4	297/493	0.93(0.82–1.05)		210/473	0.97 (0.84–1.12)	
Cancer site							
	Colon	332/626	0.94(0.84–1.06)	0.80	255/611	0.97 (0.85–1.10)	0.84
	Rectum	181/364	0.86(0.71–1.04)		140/355	0.94 (0.76–1.17)	
Stage ^e^							
	I–II	127/410	0.95(0.78–1.15)	0.90	78/396	1.09 (0.85–1.38)	0.37
	III–IV	323/446	0.95(0.84–1.08)		277/442	0.96 (0.84–1.11)	
Region ^f^							
	Northern	194/330	0.88(0.77–1.01)	0.49	158/325	0.90 (0.77–1.05)	0.31
	Central	226/432	0.96(0.82–1.13)		163/414	0.94 (0.78–1.14)	
	Southern	93/228	1.03(0.79–1.34)		74/227	1.20 (0.88–1.62)	

^a^ Excluded 24 cases with missing cause of death. ^b^ Adjusted for age, sex, stage, smoking status, BMI, site of primary tumor, year of diagnosis, and baseline diabetes. Stratified by country. ^c^ P for trend or interaction (as estimated by the likelihood ratio test). ^d^ P for trend. ^e^ Participants with missing data were not included in the analysis. ^f^ Geographic regions: Northern = Denmark; Central = UK, The Netherlands, Germany, North of France; Southern = South of France, Italy, Spain.

## Data Availability

The data that support the findings of this study are available on request from the corresponding authors. The data are not publicly available due to privacy or ethical restrictions.

## Supplementary Materials

The following are available online at https://www.mdpi.com/article/10.3390/biomedicines9111521/s1, Figure S1: Spline analysis showing the association between log-transformed blood Se concentrations and risk for overall mortality, Figure S2: Spline analysis showing the association between log-transformed blood SELENOP concentrations and risk for overall mortality, Figure S3: Spline analysis showing the association between log-transformed blood Se concentrations and risk for CRC-specific mortality, Figure S4: Spline analysis showing the association between log-transformed blood SELENOP concentrations and risk for CRC-specific mortality, Figure S5: Cumulative incidence curves of CRC-specific mortality by tertiles of prediagnostic blood Se concentrations among CRC cases in the EPIC study, Figure S6: Cumulative incidence curves of CRC-specific mortality by tertiles of prediagnostic blood SELENOP concentrations among CRC cases in the EPIC study.
