# Supplementary material for: Prediagnostic Blood Selenium Status and Mortality among Patients with Colorectal Cancer in Western European Populations
Source: Biomedicines. 2021 Oct 22;9(11):1521. doi: 10.3390/biomedicines9111521 (PMC8614984; doi:10.3390/biomedicines9111521)

SUPPLEMENTARY FIGURES

Supplementary Figure S1. Spline analysis showing the association between log transformed blood Se concentrations and risk for overall mortality; *P*-value for linearity = *0.07***.**

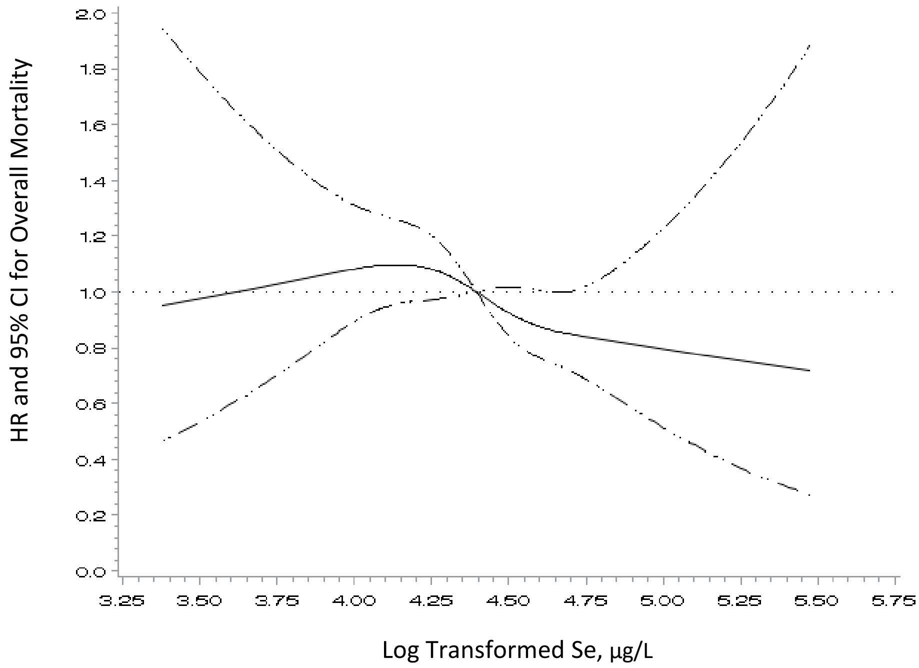

Supplementary Figure S2. Spline analysis showing the association between log transformed blood SELENOP concentrations and risk for overall mortality; *P*-value for linearity = *0.05*

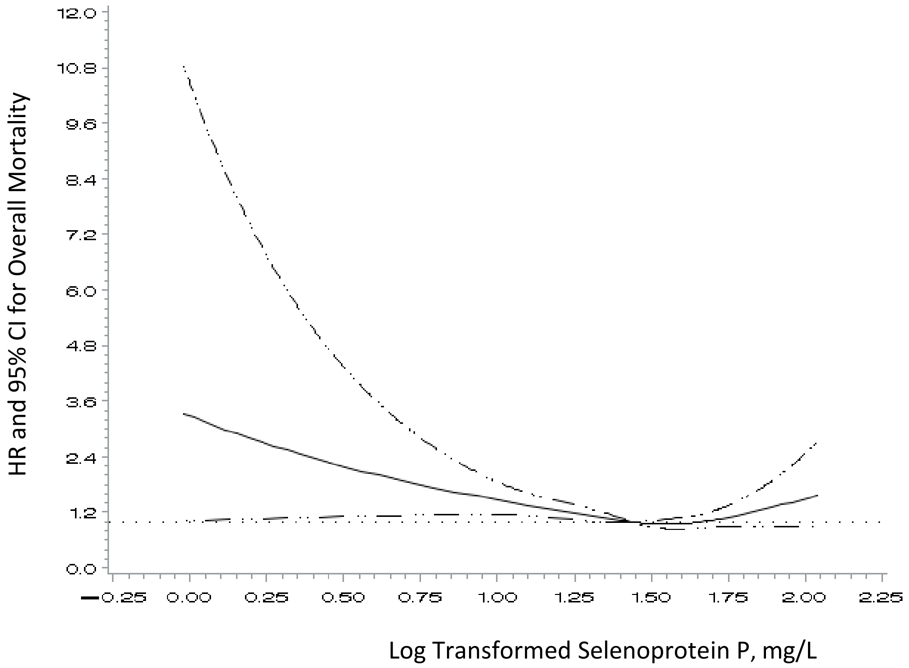

Supplementary Figure S3. Spline analysis showing the association between log transformed blood Se concentrations and risk for CRC-specific mortality; *P*-value for linearity = *0.07*

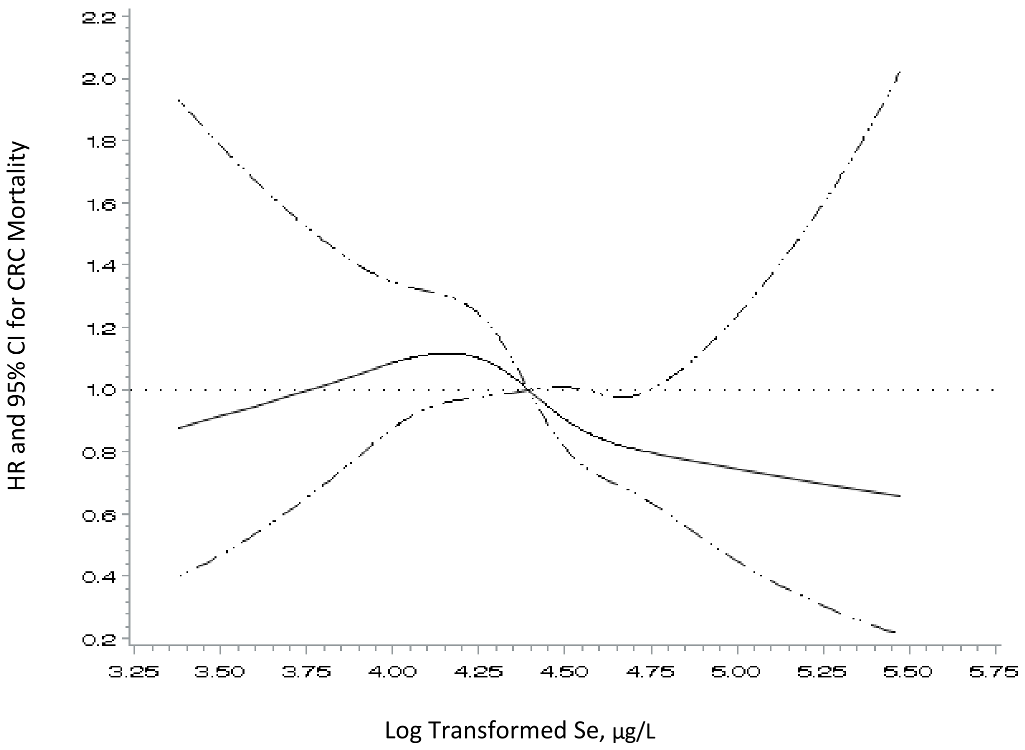

Supplementary Figure S4. Spline analysis showing the association between log transformed blood SELENOP concentrations and risk for CRC-specific mortality; *P*-value for linearity = *0.12*

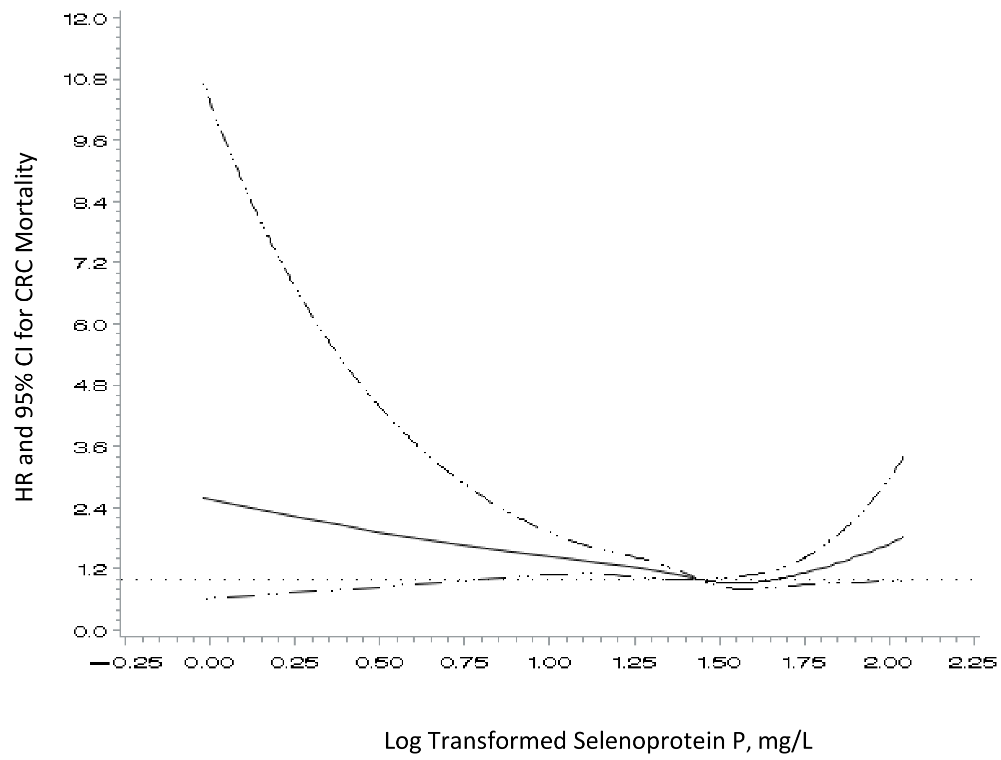

Supplementary Figure S5. Cumulative Incidence Curves of CRC-Specific Mortality by Tertiles of Prediagnostic Blood Selenium Concentrations Among CRC Cases in the EPIC study.

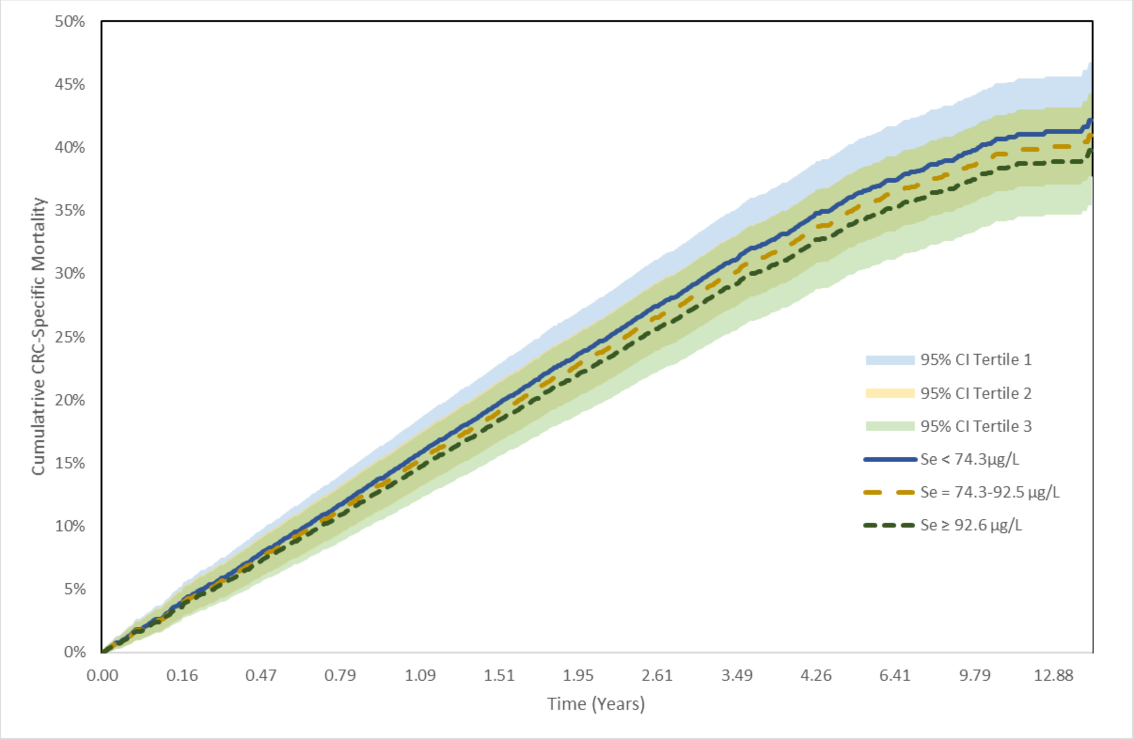

Supplementary Figure S6. Cumulative Incidence Curves of CRC-Specific Mortality by Tertiles of Prediagnostic Blood Selenoprotein P (SELENOP) Concentrations Among CRC Cases in the EPIC study.

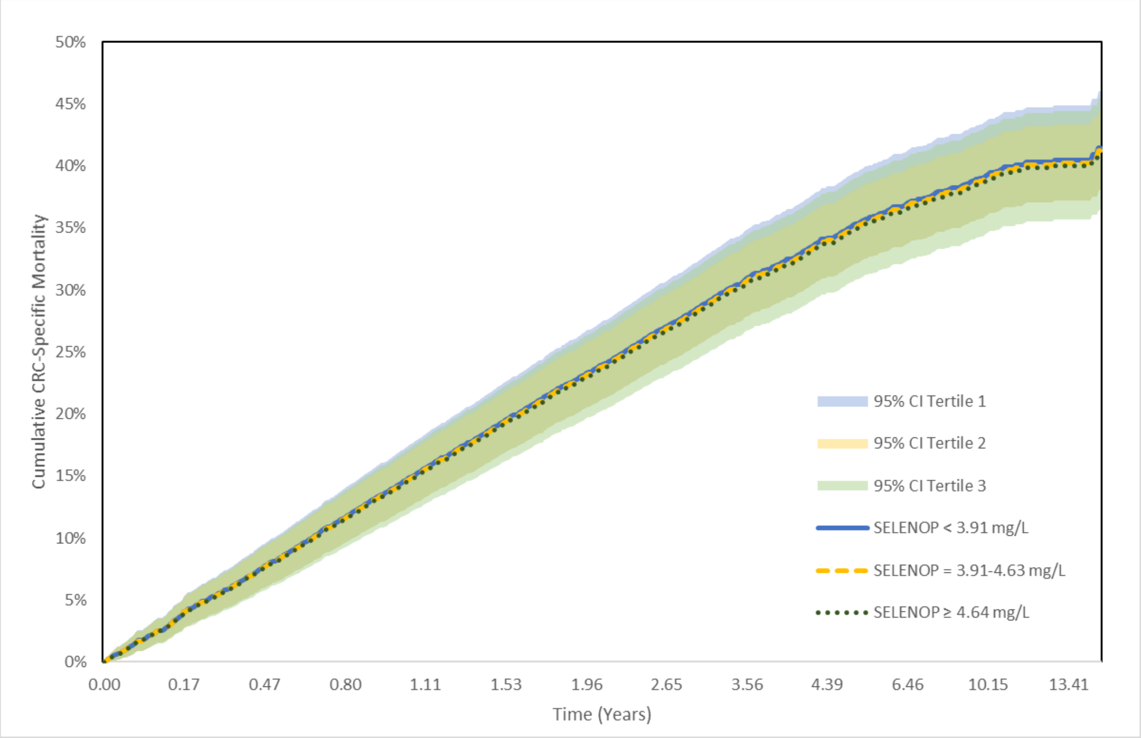

Supplement: Supplementary file 1 [file biomedicines-09-01521-s001.zip › biomedicines-1411954-supplementary.pdf]
